# Alcohol Abuse and Physical Violence towards a Partner: How Can We Attenuate the Relationship? A Study on Emotional Dysregulation in Adolescents

**DOI:** 10.3390/bs14100875

**Published:** 2024-09-27

**Authors:** Cecilia Peñacoba, Alejandro Balandin, Ana Estévez, Leticia Olave, Janire Momeñe, María Dolores Chávez-Vera, José Antonio Muñiz, Itziar Iruarrizaga

**Affiliations:** 1 Faculty of Health Sciences, University Rey Juan Carlos, 28933 Madrid, Spain; cecilia.penacoba@urjc.es (C.P.); a.balandinr@alumnos.urjc.es (A.B.); 2 Faculty of Health Sciences, University of Deusto, 48007 Bizkaia, Spain; aestevez@deusto.es (A.E.); janiremomene@deusto.es (J.M.); 3 Faculty of Health Sciences, International University of Valencia, 46002 Valencia, Spain; leticiamaria.olave@professor.universidadviu.com; 4 Faculty of Humanities and Social Sciences, Technical University of Manabí, Portoviejo 130105, Ecuador; maria.chavez@utm.edu.ec; 5 Faculty of Social Work, Complutense University of Madrid, 28040 Madrid, Spain; jomuniz@ucm.es

**Keywords:** adolescence, alcohol abuse, violence against partner, emotional dysregulation, awareness, gender

## Abstract

Background: The previous literature has revealed the relationship between alcohol abuse and violent behaviours; however, the results are not always conclusive, highlighting the need to explore other variables that allow us to establish risk profiles. Aim: The goal is to examine whether the relation between alcohol abuse and physical violence towards a partner can be influenced (moderate) by difficulties in emotional regulation. Setting: A public high school in Manabí (Ecuador). Participants: A total of 1519 high school students with ages between 14 and 18 years (mean = 15.77, SD = 1.22), with 54% (*n* = 820) being male. Main Outcome Measures: The measures we observed were alcohol abuse (i.e., frequency of alcohol abuse in the past 30 days), physical violence exercised towards a partner (Violence Received, Exercised and Perceived in Dating Relationships of Young People and Adolescents) and emotional dysregulation (Difficulties in Emotional Regulation Scale; DERS). Results: It is observed that there exists significantly higher alcohol abuse in males, regardless of their age, as well as more physical violence by adolescent males towards their partners. Direct effects of alcohol abuse on physical intimate partner violence are observed in males from the age of 16 and in females from the age of 14. Moreover, the direct effects of different emotional dysregulation strategies on physical violence depend on gender. Conclusion: The moderating effects of emotional dysregulation strategies between alcohol abuse and physical intimate partner violence are observed only in the case of adolescent females (16–17 years). In particular, emotional dysregulation variables such as non-awareness, impulse, nonacceptance, or lack of strategies interfere as moderators in the relationship between excessive alcohol abuse and physical violence towards a partner. In the case of non-awareness, contrary to the other three, when scores are low or moderate, a statistically significant relationship between alcohol abuse and violence is observed.

## 1. Introduction

Teen dating violence (TDV), recognised as an important and widespread public health problem, refers to any conduct within an intimate relationship that causes physical, psychological, or sexual harm to either partner [1]. Despite the large variability found, the previous literature indicate that around 20% of adolescents report having experienced physical and psychological TDV and 9% report having experienced sexual TDV [2,3].

Previous studies have focused on establishing risk profiles for TDV. Initial studies focused on the influence of gender and age, but did not find conclusive results. The results do, however, show a differential profile depending on these variables. Thus, while boys in earlier adolescence report more fear and injury victimisation, girls in later adolescence report more injury perpetration and suffer greater psychological consequences [2,4,5].

What appears to be a common denominator in the previous literature is that TDV entails devastating emotional and physical effects, regardless of age and gender [4]. Other socio-demographic variables have also been analysed as risk factors, such as socio-demographic and territorial level, as living in disadvantaged countries or neighbourhoods where there is a higher rate of violence may increase the likelihood of experiencing TDV [3,6].

According to the National Survey on Family Relations and Gender Violence against Women (ENVIGMU) [7], 64.9% of women in Ecuador report having experienced violence in their lifetime. Of these, 40.8% have occurred in the intimate partner sphere, with psychological violence being the most prevalent and generally becoming the step prior to physical violence [8]. It should be noted that although these data were collected on a sample of women over 15 years of age, no explicit results were obtained on the prevalence of intimate partner violence against women during adolescence.

Different theories have been proposed to explain the phenomenon of intimate partner violence in adolescence. Of these, the most widely accepted is based on the social learning theory [9], which is still very consistent with the intergenerational model of domestic violence [10]. This theory states that an aggressive and repressive interpersonal style is learned through experiences of violence in the family of origin. Parental childhood experiences of abuse are not associated with abusive behaviour towards children but are associated with other forms of violence towards partners or non-family members [11]. Such experiences of family violence in childhood may result in increased negative affectivity and emotional dysregulation [12].

There are different models of emotional regulation that are key to understanding the use of problem behaviours as a strategy to cope with unpleasant emotions. Gross’s [13] process model of emotional regulation is organised in phases, namely situation, attention, interpretation, and response, differentiating between antecedent-based and response-focused regulation. This model suggests that people can avoid situations or focus on certain aspects to regulate their emotions, although it has limitations, such as the omission of emotional acceptance, and it may promote avoidant behaviours. On the other hand, Hervás’ [14] model based on emotional processing proposes an active understanding of emotions through six phases, namely opening, attention, acceptance, labelling, analysis, and emotional modulation. This model highlights the importance of understanding and managing emotions appropriately, pointing out that substance use can be an external self-regulatory strategy to alleviate negative emotional states, as shown in studies by Fernández et al. [15] and Echeburúa [16] (1999). A recent review examines precisely the relationship between a lack of emotional regulation and substance abuse [17]. Similarly, the use of violence is related to difficulties in emotional regulation and poor coping strategies, especially in the adolescent population [18].

In this regard, results obtained in a study of gender-based violence in the province of Bolivar (Ecuador) show how excessive alcohol can be established as one of the main predictors of intimate partner aggression (IPA) [19]. Despite the established association, little research has attempted to identify the mechanisms that mediate alcohol consumption and the perpetration of IPA [20]. The identification of these variables is particularly relevant, as despite the strong association between heavy drinking and IPA, little research corroborates that heavy drinking exerts a necessary or sufficient condition for partner aggression [21], as the former does not lead to the latter in all people or circumstances, but rather alcohol abuse contributes to or facilitates the occurrence of aggression [22]. In this context, it is particularly relevant to know under which circumstances or under which personality profiles these associations are established, which would provide us with a basis for violence prevention even when excessive alcohol consumption occurs. The few existing studies on the subject indicate that a pattern of heavy drinking is more consistently associated with IPA when it is moderated by factors related to self-regulation. In this sense, the emotional self-regulation of both partners may interact with alcohol consumption and influence the risk of aggression towards the partner [22,23].

Specifically, previous studies have indicated that in men with limited access to emotional regulation strategies and greater impulse control difficulties, the association between heavy alcohol consumption and TDV was stronger [24]. Emotional dysregulation, impulsivity, and hostility are constructs that are significantly and moderately correlated with each other and are associated with the perpetration of intimate partner violence at the psychological, physical, and sexual levels. Impulsivity has been shown to influence emotional dysregulation and hostility in the case of psychological and sexual intimate partner violence, whereas in the perpetration of physical violence, it was hostility that influenced emotional dysregulation and impulsivity [23].

Going deeper into emotional dysregulation, studies that have used the Difficulties in Emotional Regulation Scale (DERS) for its measurement have observed that in the case of males, physical violence is not related to any subscale of emotional dysregulation; however, in the case of females, significant positive associations of physical violence with difficulties in impulse control, a lack of emotional awareness, limited access to emotional regulation strategies, and a lack of emotional clarity were found. In the case of psychological aggression, a positive and significant relationship with difficulties in impulse control and a lack of emotional clarity has been found in males. Among females, significant and positive relationships of psychological aggression were observed with difficulties in impulse control, difficulties in goal-directed behaviour, a lack of emotional clarity, and limited access to emotional regulation strategies [4].

**Figure 1:**
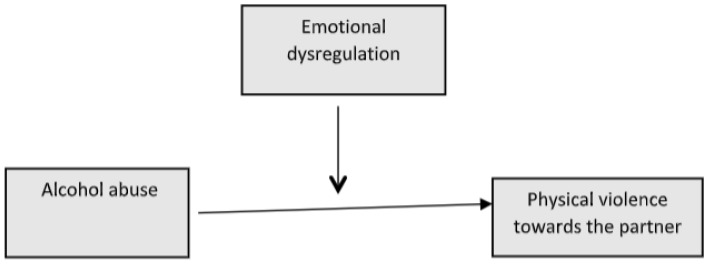
Proposed model.

As can be seen, the previous literature has established a relationship between excessive alcohol consumption and intimate partner violence [20,25,26], while the latter has been associated with certain emotional dysregulation strategies with differential profiles in men and women [22]. However, to our knowledge, little is known about the influence of emotional dysregulation strategies on the relationship between heavy drinking and physical IPA, and to what extent this influence may be gender- or age-dependent. Therefore, the main objective of the present research is to analyse the possible moderating role of emotional dysregulation strategies between excessive alcohol consumption and intimate partner violence in an adolescent population in Ecuador (see Figure 1). This possible influence will be analysed while taking into account gender and different age groups. The identification of these factors in an adolescent population can help to improve etiological models and forms of prevention and intervention that will be carried out later with much more defined risk groups.

## 2. Material and Methods

### 2.1. Design

A cross-sectional correlational study was used.

### 2.2. Participants

The sample consisted of 1519 schooled adolescents, of whom 53.9% were male (*n* = 826), aged between 14 and 18 years (M = 15.76, SD = 1.25) and belonging to twelve public education units of the different urban (63.86%; *n* = 979) and rural (36.13%; *n* = 554) socio-demographic sectors of the Portoviejo Canton of the Province of Manabí in the Republic of Ecuador.

A probabilistic method was used with stratified random sampling with proportional allocation, and the distribution was based on the weight or size within the population. Stratification was based on the number of educational units, the number of adolescents per class, urban and rural parishes, and the distribution of classes by sex. The reference population consisted of adolescents attending public schools of the urban and rural parishes of the Portoviejo Canton in the Province of Manabí (Republic of Ecuador) who belong to different socio-demographic sectors with common characteristics and constitute the object of the study. Specifically, adolescents in the tenth grade and the first, second, and third year of high school participated in the study. In order to obtain the sample, Ecuador’s official bodies were considered (National Council for the Control of Narcotic and Psychotropic Substances, 2005), which is now known as the Technical Secretariat on Drugs (SETED). The sample design and sample size were defined and calculated following the same criteria as those used in the report of the second National Survey of Secondary School Students on Drug Use (2005) of the Republic of Ecuador conducted by the National Council for the Control of Narcotic and Psychotropic Substances. These parameters used for the calculation, as referred to by the council in the report, are those applied to all studies and carried out across the continent. The target population was the fiscal educational units of adolescents in the tenth grade and the first, second, and third year of baccalaureate studies. The selection of the educational units was obtained from the database of the Ministry of Education Zonal Coordination 4 district 13D01; the educational units belong to the canton, established parish, and zone of institution (urban and rural), and support was related to the fiscal educational units and representative of different socio-demographic zones of the Canton of Portoviejo. A confidence level of 0.95 with a margin of error of 0.015 was used to calculate the sample size. Due to the sampling characteristics, a correction factor was considered due to the design effect to increase the sample size and decrease the variability of the observations; this factor was estimated at 2. Finally, the sample size was increased to compensate for a 10% possible non-response.

### 2.3. Procedure

This study was carried out with a rigorous commitment to the ethical standards governing psychological research. The study was conducted in accordance with the Declaration of Helsinki [27] and approved by the Ministry of Education of Portoviejo (Ecuador), and the academic committee of the doctoral programme in Social Work (Complutense University of Madrid, Spain) approved it, considering its scientific quality adequate and not identifying any ethical issues for its development.

Informed consent forms were submitted and signed by the parents and/or guardians of the adolescents who completed the questionnaires. They were also informed of the rules for completion, the duration and questions to be measured, the voluntary nature of the study, the confidentiality and anonymity of the data obtained, and the telephone number and e-mail addresses of the reference researchers for contacting them. Since the data collection was carried out in paper format, during the administration of the questionnaires, the researchers remained in the classroom with the students until all the completed questionnaires were returned to them. The students who collaborated in the research received a pencil and a certificate of participation as a token of appreciation. 

### 2.4. Variables and Instruments

#### 2.4.1. Alcohol Abuse

An item assessing the frequency of alcohol abuse in the past 30 days (“In the last 30 days how many days have you been drunk by drinking alcoholic beverages?”) was used with an 8-point Likert-type response format, with the scores as follows: 0 (no day), 1 (1 day), 2 (2 days), 3 (3 days), 4 (4–5 days), 5 (6–9 days), 6 (10–19 days), 7 (20–30 days). This item is extracted from the Survey on Drug Use in Secondary Education in Spain [28] (ESTUDES). The ESTUDES survey is part of a long-running series of biennial surveys conducted in Spain since 1994. Its main goal is to understand the patterns and trends in drug use among students aged 14–18 enrolled in secondary education. The survey comprises 89 questions that collect data on various aspects, including basic personal and environmental characteristics (P.1–P.17), leisure activities (P.18–P.20), lifetime, past–year, or past–month tobacco use (P.21–P.31), alcohol consumption (P.32–P.43), the use of tranquillisers, sedatives, and sleeping pills (P.44), over-the-counter tranquillisers and sedatives (P.45), and other drug types (P.46–P.61). It also covers issues experienced in the past year, family rules (P.62–P.75), drug availability (P.76–P.77), access to information (P.78–P.81), perceptions of the problem (P.82–P.83), renewed cannabis use (P.84), questions from international surveys (P.85–P.88), and suggestions for improving the questionnaire (P.89). A pilot test was conducted beforehand to assess the length and clarity of the questions.

#### 2.4.2. Intimate Partner Physical Violence

The corresponding dimension of the Scale of Violence Received, Exercised and Perceived in Dating Relationships of Young People and Adolescents—VREP [29] used. VREP allows for the evaluation of five types of violence (physical, sexual, psychological–social, psychological humiliation—coercion, and psychological control—jealousy) and three aspects of violence (exerted, received, and perceived) through 28 items. The physical violence subscale assessed in this study consists of 5 items with a Likert-type response format with 6 response alternatives (0: “Never”, 1: “Once”, 2: “2 to 5 times”, 3: “6 to 10 times”, 4: “11 to 15 times” and 5: “More than 15 times”). Some of the items on the scale are as follows: “I pinched him with the intention of hurting him” and “I have deliberately bitten or pulled his hair”. It is worth mentioning that a higher score on the items indicates a stronger presence of violence (in any of its forms), whether experienced, perpetrated, or perceived. In order to complete the scale, participants were required to have or have had at least one relationship of more than a one-month duration. Participants are asked to indicate whether the situations described have occurred or are occurring in their relationships and how often they occur. In the original study, the scale had a Cronbach’s alpha coefficient of 0.99 [29]. In this study, Cronbach’s alpha coefficient for the physical violence subscale was 0.94.

#### 2.4.3. Difficulties in Emotional Regulation

The Difficulties in Emotional Regulation Scale—DERS [30] in its Spanish version [31] by Gómez-Simón et al. (2014) was used. The scale contains 36 items with a Likert-type response scale of five options from 0 (“Almost never”) to 5 (“Almost always”). This instrument allows the assessment of six emotional regulation deficits as follows: a lack of emotional awareness (difficulties in admitting emotional states and tending to these emotions, i.e., “When I’m upset, I become embarrassed for feeling that way”), difficulties in impulse control (difficulties in controlling certain behaviours triggered by experiencing negative emotions, i.e., “When I’m upset, I become out of control”), a lack of emotional acceptance (showing a negative emotional response to a negative primary emotional stimulus, i.e., “When I’m upset, I become embarrassed for feeling that way”), interference in goal-directed behaviour (impairment or difficulty in concentration after experiencing negative emotions, i.e., “When I’m upset, I have difficulty getting work done”), a lack of emotional clarity (difficulty or lack of clarity in understanding or knowing one’s own emotions, i.e., “I have no idea how I am feeling”), and limited access to emotional regulation strategies (perceived inability to modify a negative emotional state, i.e., “When I’m upset, it takes me a long time to feel better”). A higher score implies a greater deficit in emotional regulation. In the instrument’s validation, the internal consistency of the subscales ranged from moderate to satisfactory (0.71–0.88) [31]. In the present study, the following Cronbach’s alphas were found for each of the strategies: nonacceptance of emotional responses (0.82), difficulties engaging in goal-directed behaviours (0.73), impulse control difficulties (0.80), a lack of emotional awareness (0.84), limited access to emotion regulation strategies (0.79), and a lack of emotional clarity (0.81).

### 2.5. Statistical Analysis

IBM SPSS Statistics version 27.0 [32] was used for data analysis. Univariate normality, multivariate normality, and linearity were assessed with graphical methods such as histograms and Q–Q plots. Frequency analyses were carried out in relation to alcohol abuse. Mean difference analyses were carried out for independent groups (Student’s *t*-test) on the variables under study (excessive alcohol abuse, physical violence against a partner, and emotional dysregulation strategies). Specifically, two types of analyses were carried out, one with respect to gender (males vs. females) and one with respect to age group (14–15 years vs. 16–18 years). A series of multivariate regressions were then computed with the PROCESS macro (model 1) [33]. In each regression, a combination of the independent variable (i.e., excessive alcohol abuse), the moderator (i.e., emotional dysregulation strategy), and their interaction was entered to predict the outcome (i.e., physical violence against a partner). These analyses were further conducted in four distinct subgroups, which were males aged 14–15 years, males aged 16–18 years, females aged 14–15 years, and females aged 16–18 years. In total, 24 multivariate regressions were performed (6 for each subgroup). Post hoc analyses were calculated when significant moderation was found to obtain the conditional effects of the independent variables on outcomes at different levels of the moderator. An alpha level of 0.05 was set for all analyses. 

## 3. Results

### 3.1. Differences in Excessive Alcohol Abuse, Physical Violence against Partners, and Emotional Dysregulation Strategies According to Gender and Age

Table 1 shows the frequencies and percentages for each of the alcohol consumption response options, including the median value. Table 2 presents the mean values for alcohol abuse, physical violence towards partners, and the different emotional regulation strategies, including a comparison of means according to gender and age. As can be seen in Table 1 and Table 2, males show significantly higher frequencies than females in excessive alcohol abuse (*p* < 0.001), although the effect sizes point to a weak association. Physical violence towards partners also shows significantly higher values for males (*p* = 0.04). Regarding differences by age group, statistically significant differences were observed in physical violence against partners, with higher scores in the 16–18 years of age group than in the 14–15 years of age group (*p* = 0.01).

With regard to emotional dysregulation strategies, statistically significant differences were only observed according to gender, specifically in the strategies of “lack of emotional awareness” and “difficulties engaging in goal-directed behaviours”. Thus, while in the former, higher scores are observed in males, in the latter, it is females who show greater dysregulation. Effect sizes are small in all cases.

### 3.2. Multivariate Associations and Moderation Analyses in Men

The results of the multivariate hierarchical regression analyses predicting physical violence against partners from excessive alcohol abuse, emotional dysregulation strategies, and their interaction in men are shown in Table 3. In males aged 16–18 years, a positive main effect of excessive alcohol abuse was found on physical violence against partners in all models (most were *p* < 0.001). Likewise, in that same age group (16–18 years), a significant main effect of all emotional dysregulation strategies was found on physical violence against partners, in all cases positive, except for “lack of emotional awareness”.

No direct effects were observed for excessive alcohol abuse nor for emotional dysregulation strategies in the group of men aged 14–15 years.

No interaction effects were observed in any of the age groups.

### 3.3. Multivariate Associations and Moderation Analyses in Women

The results of the multivariate hierarchical regression analyses predicting physical violence against partners from excessive alcohol abuse, emotional dysregulation strategies, and their interaction in women are shown in Table 4. In females aged 14–15 years, a positive main effect of excessive alcohol abuse was found on physical violence against partners in most models (except in the combination of alcohol abuse with “Impulse control difficulties” (*p* = 0.05) and “limited access to emotion regulation strategies” (*p* = 0.07)). None of the emotional dysregulation strategies have direct effects on this age group (14–15 years). No interaction effect is observed.

In females aged 16–18 years, a positive main effect of excessive alcohol abuse was found on physical violence against partners in all models. Likewise, in that same age group (16–18 years), a significant main effect of emotional dysregulation strategies was found on physical violence against partners, except for “lack of emotional awareness” and “lack of emotional clarity”. In all cases where the relationship is significant, it is positive. In this age group (16–18 years), four moderation effects of emotional dysregulation strategies were revealed, one in the relationship between alcohol abuse and “lack of emotional awareness” in physical violence against partners (β = −0.08, t = −3.69, *p* < 0.001, 95% CI = −0.13, −0.03), another in the relationship between alcohol abuse and “Impulse control difficulties” in physical violence against partners (β = 0.05, t = 2.27, *p* = 0.02, 95% CI = <0.01, 0.10), another one in the relationship between alcohol abuse and “Nonacceptance of emotional responses” in physical violence against partners (β = 0.07, t = 4.87, *p* < 0.001, 95% CI = 0.04, 0.11), and a final one in the relationship between alcohol abuse and “limited access to emotion regulation strategies” in physical violence against partners (β = 0.06, t = 2.65, *p* < 0.01, 95% CI = 0.01, 0.09).

As noted in Table 5, in the case of “limited access to emotion regulation strategies”, “impulse control difficulties”, and “nonacceptance of emotional responses”, the analyses of the conditional effects indicated that the strength of the (positive) relationship between alcohol abuse and physical violence towards a partner increased notably as emotional dysregulation strategies increased. In particular, the association between alcohol abuse and physical violence towards a partner was no longer significant at low levels of these emotional dysregulation strategies.

On the contrary, in the case of “lack of emotional awareness”, the association between alcohol abuse and physical violence towards a partner was only significant at low and medium levels of this emotional dysregulation strategy. That is, the greater the use of emotional awareness as an emotional regulation strategy, the greater the relationship between alcohol abuse and intimate partner violence.

## 4. Discussion

This paper aims to shed some light on the possible moderating role that emotional dysregulation strategies may have in the well-known association between alcohol abuse and physical violence towards partners in adolescence [20,25,26]. We consider it of special interest to delve deeper into the moderating variables of the relationship between alcohol abuse and physical violence in adolescents. Research interest in this population has focused on alcohol abuse at this stage of development and with the beginning of intimate partner relationships, as well as the presence of violence in these relationships [34]. Violence can be particularly severe in adolescence, especially at the beginning of intimate personal relationships [2,3].

In addition, there exists considerable literature indicating that both alcohol abuse and physical violence towards partners depend on both gender and age [35,36]. Therefore, in our aim to explore the moderating role of emotional regulation between alcohol abuse and physical violence towards partners, we will do so differentially according to the gender and age of the participants. To our knowledge, these relationships have not been explored.

Our bivariate results support the existing literature indicating a higher incidence of both problem behaviours (alcohol abuse and intimate partner violence) in men than in women. Research indicates that excessive alcohol abuse is higher in male adolescents than in female adolescents [37,38,39,40]. Our results support the higher risk profile in males, regardless of the age. With regard to physical violence towards intimate partners, our data are also in line with the previous research, which shows higher scores for men [41].

Regarding the central variable of our research, initial bivariate results indicate differences in the use of emotional dysregulation strategies between men and women. This fact has been sufficiently investigated in the previous literature [42,43]. Specifically, our findings point to a greater lack of emotional awareness in males as in previous studies [44], in line with a higher prevalence of alexithymia in males [45].

On the other hand, with respect to the difficulties engaging in goal-directed behaviours, our results, as observed in previous studies [46], show higher scores in women.

In relation to the main objective of our research, one finding of interest is that despite the introduction of the different emotional dysregulation strategies as moderating variables, there is still a direct effect of excessive alcohol consumption on physical violence in both adolescent males and females. This direct relationship holds in all cases, irrespective of the emotional dysregulation strategy and in both males and females. This fact is particularly relevant and has important practical implications. The amplifying/buffering role of emotional (dys)regulation strategies in the relationship between alcohol abuse and physical violence towards one’s partner does not negate the direct effect between these problematic behaviours highlighted in the previous literature [20,25,26].

Of particular interest in our study is the age differential between men and women, where the effect of alcohol abuse on physical violence towards partners is significant. Thus, while in male adolescents, alcohol abuse only has a direct effect on physical violence towards partners from the age of 16, in the case of females, this effect is observed at earlier stages (pre-adolescence, 14–15 years). Previous studies [47,48] have already pointed out that alcohol abuse begins in many cases in pre-adolescence, with the onset of poly-drug use patterns (including other drugs and not only alcohol) being observed from the age of 16. This alcohol abuse has been associated, in the case of men, with a marked increase in the likelihood of perpetrating a physical assault compared to non-drinkers [49]. However, there are few previous studies that delve into a differential analysis between men and women [15] and to our knowledge, there are none that also consider emotional dysregulation. This is why our findings point to the need to intervene, especially early on in alcohol abuse in adolescent women, in order to prevent physical violence against partners.

In the analysis of the effects of emotional dysregulation strategies on physical violence, the results obtained point to direct effects in both male and female adolescents. It is of additional interest that this effect is observed in both males and females in the 16–18 years age group, but not in early adolescence (14–15 years). Likewise, the results indicate that in the group of 16–18-year-old males, all emotional dysregulation strategies have a direct effect on physical violence against partners, while in females of the same age, the effect is more selective (not affecting all strategies). Thus, in the case of women, these direct effects involve maladaptive strategies such as impulsivity, which represent a key strategy in explaining dating violence, nonacceptance, a lack of goals, and a lack of strategies [50,51,52,53]. Although to our knowledge, there are no previous studies that analyse these relationships in adolescents differentiated by age and gender, our data as a whole (except in the case of “lack of emotional awareness”) indicate that better emotional regulation leads to a lower perpetration of violence, consistent with the previous literature [44,54]. According to the models of emotional regulation proposed by Gross (1999) [13] and Hervás (2011) [14], the impact of emotional regulation strategies on violent behaviours observed in adolescents can be better understood. Gross [13] (1999) classifies emotional regulation strategies as adaptive and maladaptive, such as cognitive reappraisal and emotional suppression, respectively. Hervás [14] (2011) highlights the importance of self-regulation and self-reflection in emotional regulation, especially in the component of emotional modulation. These components are particularly relevant in late adolescence given that, as mentioned, maladaptive strategies such as impulsivity or a lack of emotional acceptance in females and the combination of several strategies in males may enhance physical violence in relationships. Thus, the promotion of emotional regulation strategies would be essential to reduce these aggressive behaviours.

A particularly striking result observed in males is the effect of “lack of emotional awareness” on physical violence towards a partner. Somewhat counter-intuitively a priori, it seems that high levels of this dysregulation strategy, in contrast to the rest of the strategies, are related to less physical violence. Given the lack of previous research on this topic, some questions could be hypothesised to try to explain these results. On the one hand, it could be argued that showing an excess of emotional awareness could be detrimental, since emotions regulate people’s behaviour and their strength drives actions [55,56]. People tend to internalise and want to achieve social expectations, which when not achieved causes them to experience a series of negative feelings or emotions in search of balance in the situation. Being fully aware of and ruminating on this anguish, helplessness, or despair can sometimes lead to violent behaviour [55]. In this sense, studies analysing the different role of emotional intelligence dimensions have confirmed the not-always-healthy role of high levels of emotional attention, as opposed to repair and emotional clarity [57]. On the other hand, it is necessary to take into account the contextual variables where the present data have to be interpreted, namely the adolescence stage. Thus, it should be taken into account that this excess of emotional awareness, in the present study, occurs at a stage in which the acquisition of adaptive emotional regulation strategies is not always a reality. As is well known, emotional regulation is a complex process that is acquired from childhood, guided by adults in the family and the school environment until adolescence, where emotions are understood in a more individualised and complex way due to the development of cognitive skills [58]. Having reached this stage with better tools at the level of emotion regulation will influence experiencing greater subjective well-being, comfort or satisfaction with oneself, being able to regulate oneself better and presenting more socially appropriate behaviours, and having a lower probability of displaying problematic behaviours in adolescence and later life stages [58,59,60]. The lack of adaptive strategies for coping with various situations, enhanced by an excess of emotional awareness, could lead to the use of violence as the only option for dealing with them [24,50,51]. Along the same lines and without leaving contextual variables aside, it is known that at this stage, peer pressure and social pressure play a fundamental role as predictors of behaviour [61,62,63]. At an evolutionary stage where adaptive regulation strategies are not particularly consolidated [64] and where violence as a means of regulation is socially reinforced [65,66], it could be understood that an excess of emotional awareness is associated with a greater intensity of physical violence towards partners. Certain explanatory theories such as Bandura’s (1977) [9] social theory and processes such as the intergenerational transmission of violence [10] may contribute to the learning and maintenance of violence, which in turn may be reinforced by peers in the school environment or outside it [59,67,68,69,70].

Finally, it is relevant to point out one of the most novel results of the present study. In particular, we refer to the moderation effects found in the group of women aged 16–18 years, with regard to certain strategies of emotional dysregulation between alcohol abuse and physical violence towards partners. Specifically, “Impulse control difficulties”, “Nonacceptance of emotional responses”, and “limited access to emotion regulation strategies” are shown to be strategies that at high levels (i.e., high dysregulation) favour a significant and positive relationship between alcohol abuse and physical violence. As has been pointed out, although the role of these maladaptive strategies has been discussed in relation to physical violence and violent behaviour, bidirectional relationships have been explored, especially in relation to impulsivity [71,72], without considering their possible moderating role and without delving into the differential effects according to gender or age. Given the relevance that these results may have for personalised treatments from models of psychological flexibility [73], it would be of interest to conduct further studies that delve into these relationships. In the case of “lack of emotional awareness”, the apparent counter-intuitive effect, previously mentioned regarding its direct effect on physical violence in men, is once again observed, in this case in relation to its moderating role. In the absence of previous studies in this regard, as mentioned above, the results obtained (direct effect on males, moderating effect on females) point to the need for a differential analysis of the “emotional awareness” strategy due to its complexity and the adaptive and maladaptive results to which it may be associated, probably explained by its combination with other contextual variables or other regulatory mechanisms.

### Practical Implications

Despite the moderating influence of emotional regulation, a direct relationship persists between alcohol consumption and violent behaviour toward one’s partner. Problematic alcohol consumption is considered one of the main risk factors for violence [74,75], while the sale of alcohol has shown a positive and significant correlation with general homicide rates in several countries, such as those in Europe and India [76,77,78]. According to the WHO [79] (2009), interventions aimed at preventing alcohol-related violence could contribute to the reduction in various forms of violence, including intimate partner violence, child abuse, and suicide. In the systematic review by [80], which analysed 13 prevention programmes for interpersonal violence occurring under the influence of alcohol, it was highlighted that interventions targeting the perpetrators of violence (especially males), particularly those with a family-focused approach, have proven more effective in reducing intimate partner violence. This suggests that addressing alcohol consumption in this context could be a useful strategy. On the other hand, the systematic review conducted by McMurran et al. (2011) [81], aimed at determining the best interventions to prevent violence under alcohol consumption by women, found that some studies suggested potential negative effects of these interventions, while others showed short-term benefits (up to 6 months), but without lasting effects. Additionally, it was suggested that women may experience emotional issues leading to excessive alcohol consumption, and interventions that induce negative emotions could increase emotional distress, thereby exacerbating drinking behaviour and the likelihood of alcohol-related offences. Regarding types of interventions, it seems that those carried out online may be effective in reducing alcohol-related intimate partner violence in young people and adolescents [82,83] though further research is needed to obtain conclusive results [84].

Moreover, given the important role of emotional regulation as a moderating variable between alcohol consumption and violence, there is a clear need to implement strategies and programmes that provide tools for improving emotional management. In this regard, prevention programmes in schools, such as the RULER program [85,86] and INTEMO [87,88], have shown positive results in terms of effectiveness. It is crucial to strengthen those elements related to emotional intelligence that act as protective factors against violent behaviour, such as emotional management and empathy [89].

This study has some limitations that should be mentioned. First, the data are based on a cross-sectional design, so it is not possible to establish a cause-and-effect relationship. Second, adolescents that participated were recruited voluntarily in a single province, which could be limiting representativity; therefore, generalisation of the data to the general adolescent population could be compromised. Another limitation is the reliability of data obtained through self-reported measures. Specifically, when these instruments are used to assess negative variables (alcohol abuse, physical violence towards partners), participants are more likely to be biassed in their responses. Finally, although we have included important psychological mechanisms in intimate partner violence (i.e., alcohol abuse, emotional dysregulation), the list is not complete. Thus, given that the highest percentage of variance explained in our research is 30%, it would be of interest for future research to explore the role of other relevant psychosocial variables such as self-esteem, social support, self-efficacy, empathy, social skill deficits, or attachment.

Despite the above limitations, the results of our study provide new data to understand the complex relationship between alcohol abuse and physical violence towards partners, considering the role of non-adaptive emotional regulation processes. The data highlight the need to personalise treatments and/or preventive measures according to gender and age in the adolescent population.

Thus, in the case of male adolescents, the critical age is set to 16 years of age, where both alcohol abuse and emotional dysregulation strategies seem to play a significant role in physical violence towards partners. In the case of female adolescents, two differential profiles can be observed depending on their age. In the early stage (14–15 years), a significant effect of alcohol abuse on intimate partner violence is observed, but from the age of 16, in addition to alcohol, emotional dysregulation strategies play a direct effect and moderation effects are also observed in the alcohol abuse–physical violence relationship depending on the levels of emotional dysregulation.

Finally, emotional awareness does not seem a priori to be an adaptive strategy in this context. We expect that the present study will inspire future similar investigations including these and other important psychological processes, such as self-efficacy, coping, and acceptance, to name some examples. It is necessary, given the high prevalence of violent behaviour in the adolescent population [90,91,92] to increase the body of knowledge in this regard in order to design and carry out prevention and intervention programmes with groups of adolescents at risk of carrying out this type of violent behaviour.

## Figures and Tables

**Table 1:** Excessive alcohol abuse. Analysis based on gender and age.

	0	1	2	3	4	5	6	7	Median
	Ningún Día	1 Day	2 Days	3 Days	4–5 Days	6–9 Days	10–19 Days	20–30 Days	
	*n* (%)	*n* (%)	*n* (%)	*n* (%)	*n* (%)	*n* (%)	*n* (%)	*n* (%)	Median
Men (*n* = 826)	429 (52.2%)	152 (18.3%)	110 (13.2%)	55 (6.5%)	41 (5%)	16 (2%)	12 (1.5%)	11 (1.3%)	0
Women (*n* = 693)	454 (65.4%)	109 (15.8%)	48 (7%)	36 (5.2%)	21 (3%)	18 (2.6%)	3 (0.4%)	4 (0.6%)	0
Chi square = 38.114, *p* < 0.001; Cramer’s V = 0.159, *p* < 0.001									
14–15 years (*n* = 643)	386 (60.2%)	104 (16.1%)	68 (10.5%)	33 (5.2%)	27 (4.2%)	12 (1.9%)	6 (0.9%)	7 (1.1%)	0
16–18 years (*n* = 876)	498 (56.9%)	158 (18%)	90(10.3%)	56 (6.4%)	35 (4%)	22 (2.5%)	9 (1%)	8 (0.9%)	0
Chi square = 3.303, *p* = 0.856; Cramer’s V = 0.047, *p* = 0.856									

**Table 2:** Excessive alcohol abuse, physical violence against partners, and emotional dysregulation strategies. Analysis based on gender and age.

	Gender					Age				
	Men	Women				14–15 Years	16–18 Years			
	Mean (SD)	Mean (SD)	t	*p*	D	Mean (SD)	Mean (SD)	t	*p*	D
Excessive alcohol abuse	1.12 (1.58)	0.77 (1.37)	4.63	<0.001	0.23	0.92 (1.48)	0.99 (1.51)	−0.88	0.37	0.04
Physical violence against partners	1.83 (3.26)	1.51 (3.19)	1.97	0.04	0.09	1.45 (3.08)	1.85 (3.33)	−2.37	0.01	0.12
Awareness	19.70 (5.18)	18.47 (5.20)	4.58	<0.001	0.23	19.24 (5.32)	19.05 (5.15)	0.71	0.47	0.03
Impulse	14.77 (4.74)	14.72 (5.34)	0.18	0.85	<0.01	14.77 (5.06)	14.74 (5.00)	0.11	0.91	<0.01
Nonacceptance	15.81 (6.54)	16.50 (7.25)	−1.93	0.05	0.09	16.15 (7.06)	16.11 (6.75)	0.11	0.91	<0.01
Goals	13.35 (3.66)	13.86 (4.06)	−2.57	0.01	0.13	13.68 (4.01)	13.51 (3.75)	0.79	0.42	0.04
Clarity	13.26 (3.21)	13.08 (3.79)	1.00	0.31	0.05	13.13 (3.38)	13.22 (3.57)	−0.48	0.62	0.02
Strategies	16.56 (4.97)	16.93 (5.74)	−1.32	0.18	0.06	16.76 (5.43)	16.71 (5.27)	0.18	0.85	<0.01

D: Cohen’s d. Awareness: lack of emotional awareness. Impulse: impulse control difficulties. Nonacceptance: nonacceptance of emotional responses. Goals: difficulties engaging in goal-directed behaviours. Clarity: lack of emotional clarity. Strategies: limited access to emotion regulation strategies.

**Table 3:** Multivariate hierarchical regression analyses predicting physical violence against partner from excessive alcohol abuse, emotional dysregulation strategies, and their interaction (in men).

	Men 14–15 Years							Men 16–18 Years						
	*R*	*F*	*p*	Beta	*t*	*p*	95% CI	*R*	*F*	*p*	Beta	*t*	*p*	95% CI
	0.07	0.51	0.63					0.20	7.08	<0.001				
Alcohol abuse				−0.09	−0.54	0.58	−0.44, 0.25				0.36	3.78	<0.001	0.17, 0.55
Awareness				−0.06	−1.18	0.23	−0.17, 0.04				−0.07	−2.53	0.01	−0.13, −0.02
Interaction				<0.01	0.17	0.85	−0.06, 0.07				0.01	0.90	0.36	−0.02, 0.05
	0.03	0.11	0.95					0.28	14.11	<0.001				
Alcohol abuse				−0.09	−0.56	0.57	−0.44, 0.24				0.33	3.45	<0.001	0.14, 0.52
Impulse				0.01	0.18	0.85	−0.11, 0.13				0.16	5.24	<0.001	0.10, 0.22
Interaction				<0.01	0.04	0.96	−0.07, 0.07				<0.001	0.05	0.96	−0.04, 0.04
	0.06	0.46	0.70					0.21	8.03	<0.001				
Alcohol abuse				−0.10	−0.58	0.55	−0.45, 0.24				0.36	3.66	<0.001	0.17, 0.56
Nonacceptance				0.01	0.31	0.75	−0.07, 0.10				0.06	2.80	0.005	0.02, 0.11
Interaction				0.02	0.97	0.32	−0.02, 0.07				−0.02	−1.18	0.24	−0.05, 0.01
	0.07	0.66	0.57					0.22	9.11	<0.001				
Alcohol abuse				−0.10	−0.58	0.56	−0.45, 0.24				0.37	3.90	<0.001	0.19, 0.57
Goals				0.05	0.79	0.42	−0.08, 0.20				0.14	3.20	0.001	0.05, 0.22
Interaction				0.05	1.07	0.28	−0.04, 0.14				−0.04	−1.19	0.23	−0.09, 0.02
	0.06	0.40	0.74					0.19	6.64	<0.001				
Alcohol abuse				−0.06	−0.37	0.70	−0.41, 0.28				0.34	3.45	<0.001	0.15, 0.54
Clarity				−0.06	−0.69	0.48	−0.25, 0.12				0.11	2.42	0.01	0.02, 0.19
Interaction				−0.04	−0.73	0.46	−0.17, 0.07				<0.01	−0.07	0.94	−0.06, 0.06
	0.04	0.19	0.89					0.26	11.86	<0.001				
Alcohol abuse				−0.10	−0.59	0.54	−0.46, 0.24				0.31	3.16	<0.01	0.12, 0.50
Strategies				0.03	0.52	0.59	−0.08, 0.15				0.13	4.60	<0.001	0.08, 0.19
Interaction				<0.01	0.23	0.81	−0.06, 0.08				<0.01	0.09	0.92	−0.04, 0.04

Awareness: lack of emotional awareness. Impulse: impulse control difficulties. Nonacceptance: nonacceptance of emotional responses. Goals: difficulties engaging in goal-directed behaviours. Clarity: lack of emotional clarity. Strategies: limited access to emotion regulation strategies.

**Table 4:** Multivariate hierarchical regression analyses predicting physical violence against partners from excessive alcohol abuse, emotional dysregulation strategies, and their interaction (in women).

	Women 14–15 Years							Women 16–18 Years						
	*R*	*F*	*p*	Beta	*t*	*p*	95% CI	*R*	*F*	*p*	Beta	*t*	*p*	95% CI
	0.13	1.83	0.14					0.24	7.57	<0.001				
Alcohol abuse				0.32	2.30	0.02	0.04, 0.60				0.38	3.33	<0.001	0.15, 0.60
Awareness				<0.01	0.14	0.88	−0.06, 0.07				−0.04	−1.48	0.13	−0.10, 0.01
Interaction				0.02	0.95	0.34	−0.02, 0.08				−0.08	−3.69	<0.001	−0.13, −0.03
	0.14	2.14	0.09					0.30	12.79	<0.001				
Alcohol abuse				0.27	1.93	0.05	<0.01, 0.55				0.21	1.91	0.04	<0.01, 0.43
Impulse				0.04	1.34	0.17	−0.02, 0.10				0.15	5.21	<0.001	0.09, 0.21
Interaction				<0.01	−0.09	0.92	−0.05, 0.04				0.05	2.27	0.02	<0.01, 0.10
	0.12	1.81	0.14					0.31	13.34	<0.001				
Alcohol abuse				0.31	2.19	0.02	0.03, 0.60				0.26	2.36	0.01	0.04, 0.48
Nonacceptance				0.01	0.54	0.58	−0.03, 0.06				0.06	3.04	<0.01	0.02, 0.11
Interaction				−0.01	−0.77	0.44	−0.04, 0.02				0.07	4.87	<0.001	0.04, 0.11
	0.12	1.60	0.18					0.18	4.49	<0.01				
Alcohol abuse				0.30	2.18	0.02	0.03, 0.58				0.32	2.83	<0.01	0.10, 0.55
Goals				−0.01	−0.32	0.74	−0.10, 0.07				0.08	2.14	0.03	<0.01, 0.16
Interaction				−0.01	−0.37	0.70	−0.08, 0.05				0.05	1.63	0.10	−0.01, 0.12
	0.13	1.88	0.13					0.14	2.73	0.04				
Alcohol abuse				0.32	2.31	0.02	0.04, 0.60				0.29	2.47	0.01	0.06, 0.52
Clarity				<0.01	−0.02	0.97	−0.09, 0.09				0.04	1.08	0.27	−0.03, 0.13
Interaction				−0.03	−1.02	0.30	−0.11, 0.03				<0.01	−0.22	0.82	−0.07, 0.06
	0.13	1.89	0.13					0.24	7.56	<0.001				
Alcohol abuse				0.25	1.76	0.07	−0.02, 0.53				0.24	2.15	0.03	0.02, 0.47
Strategies				0.02	0.93	0.34	−0.03, 0.08				0.08	2.94	<0.01	0.02, 0.14
Interaction				0.01	0.47	0.63	−0.03, 0.05				0.06	2.65	<0.01	0.01, 0.09

Awareness: lack of emotional awareness. Impulse: impulse control difficulties. Nonacceptance: nonacceptance of emotional responses. Goals: difficulties engaging in goal-directed behaviours. Clarity: lack of emotional clarity. Strategies: limited access to emotion regulation strategies.

**Table 5:** Conditional effects of alcohol abuse on physical violence (towards a partner) at values of emotional dysregulation strategies (women, 16–18 years).

Strategies	Beta (Alcohol Abuse)	*t*	*p*	95% CI	Awareness	Beta (Alcohol Abuse)	t	*p*	95% CI
−5.58	−0.07	−0.41	0.67	−0.41, 0.26	−5.39	0.84	4.59	<0.001	0.48, 1.20
−0.58	0.21	1.83	0.06	−0.01, 0.44	−0.39	0.41	3.56	<0.001	0.18, 0.64
5.57	0.56	3.59	<0.001	0.25, 0.87	5.60	−0.09	−0.60	0.54	−0.40, 0.21
Impulse	Beta (alcohol abuse)	*t*	*p*	95% CI	Nonacceptance	Beta (alcohol abuse)	*t*	*p*	95% CI
−4.50	−0.04	−0.27	0.78	−0.37, 0.28	−7.10	−0.29	−1.79	0.07	−0.61, 0.02
−0.50	0.18	1.62	0.10	−0.03, 0.41	−2.10	0.09	0.83	0.40	−0.13, 0.33
5.49	0.53	3.14	<0.01	0.19, 0.86	7.89	0.88	5.35	<0.001	0.55, 1.20

Strategies: limited access to emotion regulation strategies. Awareness: lack of emotional awareness. Impulse: impulse control difficulties. Nonacceptance: nonacceptance of emotional responses.

## Data Availability

Data are available in a publicly accessible repository. The original data presented in the study are openly available in FigShare at https://doi.org/10.6084/m9.figshare.27098200.v1.
